# Assessing Improvement in Quality of Life and Patient Satisfaction following Body Contouring Surgery in Patients with Massive Weight Loss: A Critical Review of Outcome Measures Employed

**DOI:** 10.1155/2013/515737

**Published:** 2013-07-01

**Authors:** Shehab Jabir

**Affiliations:** St. Andrews Centre for Plastic Surgery and Burns, Broomfield Hospital, Chelmsford, Essex CM1 7ET, UK

## Abstract

Body contouring following massive weight loss is a rapidly expanding field in plastic surgery. However, healthcare payers are reluctant to fund such procedures, viewing them as purely cosmetic. This has resulted in a flurry of studies assessing quality of life (QoL) and patient satisfaction following body contouring surgery in this cohort of patients to establish an evidence base to support the idea that body contouring is as much (or even more) a functional procedure as it is cosmetic. However, the methods employed in these studies are seldom ideal, and hence the conclusions are unreliable. The gold standard to assess QoL and patient satisfaction is to use patient specific psychometrically validated patient reported outcome (PRO) measures. Developing such measures consists of a three-step process which includes a review of the current literature, qualitative patient interviews to determine what patients consider the most important, and expert opinion. This study aims to appraise the currently available literature on assessment of QoL and patient satisfaction in body contouring surgery patients. This will hopefully provide an understanding of methodological weaknesses in current studies and inform future investigators of the design of ideal instruments for assessing QoL and patient satisfaction in body contouring patients.

## 1. Introduction

Body contouring surgery has undergone a rapid expansion in the last decade, becoming one of the fastest growing areas within plastic surgery. As the number of obese individuals continues to increase, bariatric surgery has come to the fore as the method of choice for rapidly losing excess weight with approximately one quarter of patients opting for bariatric surgery [1]. However, when a previously obese or morbidly obese individual loses a massive amount of weight, it results in cutaneous contour deformities on various parts of the body. These cutaneous deformities may then lead to psychological distress as well as functional problems, offsetting the positive benefits brought about by weight loss surgery [2–4]. Hence body contouring surgery would intuitively appear to be the next step in rehabilitating the obese patient with massive weight loss (MWL).

However, it has been a challenge to convince healthcare payers of the importance of body contouring procedures to the overall outcome of massive weight loss patients. Whereas funding for bariatric surgery has become easier to obtain due to mounting evidence of its benefits on the health of obese individuals and the economic implications of this benefit, it has yet to filter down to body contouring surgery. Most healthcare systems still consider body contouring to be a cosmetic procedure and are reluctant to fund such operations. This in turn has led to a number of studies addressing body contouring surgery following massive weight loss in patients who have already had bariatric surgery. The primary areas of focus of this research can be divided into two: (1) surgical outcomes in terms of procedure related complications and (2) the quality of life (QoL) and psychosocial outcome of body contouring in massive weight loss patients. In terms of procedure related complications, it has been shown that as operator experience increases, complication rates drop [5, 6]. However, the second category of research, that is, QoL and psychosocial outcome, is far more important from a funding perspective. If we are able to demonstrate that body contouring surgery results in a significant improvement of the patient's QoL and psychosocial state, then it seizes to be viewed as a purely cosmetic procedure and may make it even obligatory for healthcare payers to fund body contouring surgery as the completion step to rehabilitating the obese patient.

The gold standard for measuring the impact of body contouring surgery on massive weight loss individuals is to use patient-specific, well-constructed psychometrically validated patient reported outcome (PRO) measures (also known as instruments). PRO instruments are any report of the status of a patient's condition that comes directly from the patient without interpretation by a surgeon or other healthcare professional [7]. These measures usually consist of a number of sections which are designed to include key aspects of a conceptual framework. The conceptual framework explicitly defines the concepts measured by the instrument in a diagram that presents a description of the relationships between items, domain (subconcepts), and concepts measured and the scores produced by a PRO instrument [7]. 

 A systematic review of PRO instruments to measure quality of life and patient satisfaction following body contouring surgery was undertaken by Reavey et al. They identified five PRO measures with varying psychometric validity: one general plastic surgery (DAS 59), three breast reduction (BRASSQ, BRS, Breast-Q), and one liposuction instrument (FQAD). Following this, Reavey et al. called for the development of new PRO measures specific to this population [8]. The development of PRO measures consists of a thorough review of currently available literature, qualitative patient interviews to determine what patients consider most important, and expert opinion [9]. A comprehensive understanding of issues that are critical to patients can be obtained from these three sources of information which may then help to inform development of scales and items for inclusion in a new PRO instrument. The aim of this study is to review the methods of assessing QoL and patient satisfaction in postbody contouring MWL patients. This would enable us to delineate what measures have already been employed to assess QoL and patient satisfaction in this domain and where improvements are necessary. It is hoped that by doing so, we would be fulfilling another step toward developing new specific PRO instruments for this fast growing population of patients.

## 2. Methods

An attempt was made to make the search for the literature as systematic as possible. Hence, the PRISMA statement for systematic reviews was adhered to [10]. Items 12–16 of the PRISMA statement were not applicable to this study as it was not possible to undertake quantitative data synthesis due to wide heterogeneity in the reported outcome measures.

 The predetermined inclusion and exclusion criteria for this review were used to determine eligibility of a study to be included in the review.

Inclusion criteria include the following:

- the study population was restricted to massive weight loss patients;
- patients included in the study had either one or more of what is considered a body contouring procedure (e.g., abdominoplasty, panniculectomy, thoracoplasty, brachioplasty, mastopexy/plasty, thigh lift, etc.);
- a clearly defined method of measuring improvement in quality of life and/or psychosocial function;
- english language publication.

Exclusion criteria include the following:

- Studies measuring only overall “satisfaction” with body contouring procedures.
- Studies measuring the desire for body contouring in postmassive weight loss patients who believed they may attain some functional/psychosocial benefits from body.

The literature search was then carried out on Medline, Embase, CINAHL, PsycINFO, Google Scholar, and the Cochrane databases from inception till March 2013 for studies on the topic of improvement in quality of life and psychosocial function following body contouring surgery in massive weight loss patients. The keywords used are shown in Table 1. The terms in each column were combined with the Boolean operator “OR” while terms between columns were combined with “AND.” The output was limited to citations in the English language. Reference lists of identified studies were then hand searched for additional reports.

The title and abstract of all identified studies were examined. In cases where suitability of a study for inclusion in the review was unclear, the entire paper was obtained and assessed for suitability. Data was extracted onto a Performa which included the data categories listed.

Demographics include the following:

- mean age of study subjects;
- number of subjects;
- gender distribution, male: female;
- BMI postbariatric surgery/postmassive weight loss.

Preoperative characteristics (i.e., prior to body contouring surgery but post-bariatric/massive weight loss):

- time period between bariatric surgery and body contouring surgery;
- instrument for measurement of quality of life (QoL) and QoL as per that particular instrument of prebody contouring;
- instrument for measurement of psychosocial function and psychosocial function prebody contouring;
- body contouring procedures performed.

Postoperative characteristics (i.e., following body contouring surgery):

- QoL following body contouring surgery;
- psychosocial function following body contouring surgery;
- length of follow-up postbody contouring surgery.

Other considerations:

- psychometric assessment of tools employed;
- validity and test-retest reliability.

## 3. Results

**Figure 1:**
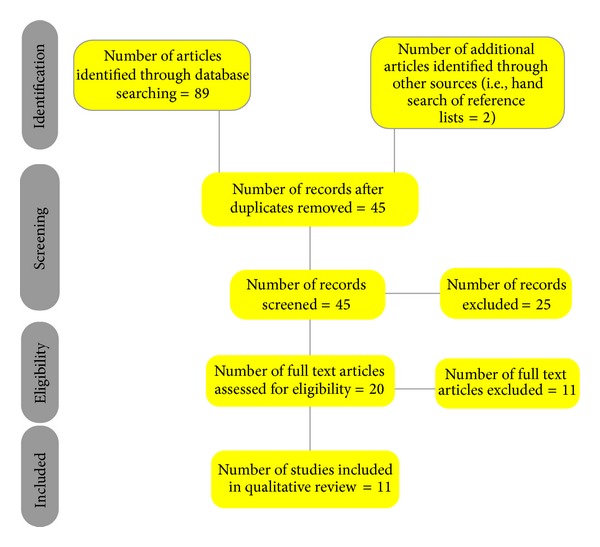
Systematic review flow diagram.

The search retrieved a total of 89 studies. Following removal of duplicates, 45 studies remained. 25 studies were excluded following screening of the title and abstract. The entire papers of the remaining 20 articles were reviewed to establish suitability for inclusion. 11 studies were excluded as they did not meet the eligibility criteria leaving 9 studies for inclusion. The reference lists of these 9 studies were then hand searched to identify any further studies. Two studies were identified in this manner, resulting in a total of 11 studies for inclusion in the review. A flow diagram of the search strategy is provided in Figure 1.

A summary of the characteristics of each study is provided in Table 2. Due to the wide heterogeneity in study design and instruments employed to measure psychosocial function and quality of life in body contouring patients, a narrative review of all studies included was undertaken.

### 3.1. Lazer et al. [11]

Two questionnaires that had been designed specifically for the study yet had not been validated were administered to 41 patients. The first tool was a three-item subjective questionnaire that assessed:

1. the patient's most problematic body areas after weight loss: abdomen, breasts, thigh, and/or arms;
2. consequences on QoL of abdominal skin overhanging: current life, dressing, aesthetics, psychological status, and/or sexual relations;
3. ranking the effects of abdominoplasty on each of the previously mentioned QoL areas as: very good, good, average quite bad, or bad.

The second tool was a nine-item questionnaire designed by a trained psychologist to assess psychological status. The questions in this tool were as follows:

1. How do you feel today?
2. What is your opinion of your abdominoplasty?
3. Are the results in accordance with your expectations?
4. Are the scars a problem for you?
5. Do you like your new body?
6. Do other people regard you differently now?
7. Has your daily life improved?
8. What if abdominoplasty had been contraindicated?
9. If you had to do it over again, would you undergo abdominoplasty?

The first questionnaire was administered once to assess preabdominoplasty body perception and QoL and for a second time to assess postbody contouring perception of improvement. The second tool was administered once to assess psychological status with all data being collected after an average follow-up period of 57.7 months (range 41 to 80 months).

In terms of the 5 areas of QoL assessed, aesthetics was the primary area of concern with 38 patients stating that they considered the abdomen an area of concern affecting their QoL prior to abdominoplasty. The next most concerning areas in terms of QoL were psychological status (36), dressing (33), sexual relations (27), and current life (15). After abdominoplasty, patients evaluated their QoL as good or very good in all of the above domains.

The second tool to assess psychological status following abdominoplasty again seemed to suggest that abdominoplasty did have a positive impact on their psychological status with 84.6% saying their daily life improved following the procedure. The results of the psychological questionnaire are presented in Table 3.

### 3.2. Song et al. [12]

Assessed body image, quality of life, and other measures in a prospective manner using a number of different instruments as shown in Table 4. A description of each of these instruments is provided in Table 5. Outcomes were assessed immediately prior to body contouring surgery, 3 months, and then 6 months after body contouring surgery. We will restrict ourselves here to the outcomes of body image and QoL. The study included a total of 18 patients, 16 females, and 2 males.

At 3 months, body image and satisfaction as measured by the BISA tool improved significantly (*P* < 0.01). The improvement remained stable at 6 months.

In terms of QoL measured by the HR-QoL, the mean score prebody contouring at 3 months postbody contouring and at 6 months postbody contouring remained around the same mark and there was not a statistically significant improvement. On the other hand, the PBSQoL scores which were collated from 13 patients however revealed a statistically significant improvement in the quality of life. 

### 3.3. Cintra et al. [13]

Assessed quality of life in 16 female patients following abdominoplasty after massive weight loss. A validated tool known as the Adaptive Operationalized Diagnostic Scale (AODS)—as described below—was used to assess QoL.

*Description of AODS Used by Cintra et al. to Assess QoL*

  AODS: A 31-item instrument consisting of 4 domains: affectivity/personal relations, productivity, social/cultural performance, and organic/somatic health. Together they evaluate physical and mental health, social adjustment, body image, self-concept, self-esteem, and mood and feelings. Results are summarized in five levels of adaptation from good (level 1) to very severe maladaptation (level 5) for each domain and as a final score for a complete test.

The patients were questioned by a trained psychologist using this tool after an interval of approximately 1–3 years following abdominoplasty. The best overall response corresponded to the social and cultural domain, where 81.3% of patients had good adaptation (level 1). For the other three domains, results were remarkably similar with 62.5% of the tests displaying the highest value of adaptation (level 1) and few complete failures. Final scoring for the complete test is demonstrated in Table 6.

In terms of specific subtopics in the domains sited in Table 5, points of note include the fact that 87.5% had a very good self-image, 87.5% displayed adequate self-esteem, and around 68.8% noticed a better sex life after abdominoplasty.

### 3.4. Stuerz et al. [14]

Carried out a prospective study including 31 postbody contouring patients with a control group of 26 patients who had undergone gastric banding and who had lost weight but who had not undergone body contouring surgery for comparison. The instruments used in this study are listed in Table 7 and were administered to the body contouring group 1 day before body contouring surgery and then at 3 and 12 months after the procedure.

In terms of body image which was assessed via the Strauss and Appelt's Questionnaire, there were improvements in all four areas listed in Table 7 with a statistically significant improvement (*P* < 0.001) in the attractiveness/self-esteem subscale in the surgery group compared with the control group. However, no significant difference was detected on the “Emphasis on attractiveness” subscale of the Body Perception Questionnaire by Paulus between the two groups. Furthermore, no change was detected in terms of life satisfaction (assessed via the Life Satisfaction Questionnaire) or anxiety and depression between the two groups (assessed via the Hospital Anxiety and Depression Scale).

The author's General Questionnaire after surgery revealed that abdominoplasty resulted in a change in leisure activities in 21 patients, reduced inhibitions in 20 patients, and improved sexual relationships in 27 patients. 

### 3.5. Pecori et al. [15]

Studied body image issues in four groups of patients who are described in Table 8.

The two groups particularly relevant to our purposes are the POST-A group and POST-B group. Body image in each of the groups was evaluated by means of the Body Uneasiness Test (BUT), a self-administered questionnaire which had displayed satisfactory test-retest reliability and internal consistency in previous studies. A description of the domains assessed by the BUT questionnaire is provided in the description of the two parts of the body uneasiness test used by Pecori et al. below.

*Body Uneasiness Test (Divided into Two Parts)*

 Part 1: explores body-related and shape-related psychopathology. Results are expressed in both a combined Global Severity Index and in scores of 5 subscales:
  1. weight phobia (fear of being or becoming fat);
  2. body image concerns (overconcerned with physical appearance);
  3. avoidance;
  4. compulsive self-monitoring (rituals involving checking physical appearance);
  5. depersonalization (feelings of detachment or estrangement from one's body).
 Part 2: indicates dissatisfaction with the overall body shape and with the different parts of one's body. Results are expressed in two domains: Positive Symptom Total and Positive Index Distress Symptom.

Table 9 demonstrates the mean scores with the standard deviation for the POST-A and POST-B groups for parts 1 and 2 of the BUT questionnaire. There is a clear improvement in all domains in the POST-B group (i.e., postobese women at >2 years after BPD who have undergone cosmetic surgery) compared to the POST-A group (i.e., postobese women at 2 years after BPD requiring cosmetic surgery). No statistical significance testing was carried out by the authors.

### 3.6. Lanier et al. [16]

 50 patients (28 women and 22 men) underwent a variety of body contouring procedures. Following these procedures, a questionnaire was administered to the patients asking the following questions:

1. Did the surgical procedure enable you to feel better about yourself in regard to the way you look and feel?
2. Did the operative procedure provide you an incentive to maintain your weight reduction goal?

Forty-eight of the 50 patients returned the questionnaire (96% response rate). Thirty-nine patients (82%) answered “Yes” when asked whether plastic surgery after weight reduction had improved their self-esteem. Thirty-four patients (72%) stated that the body contouring surgery did not provide an incentive to maintain their weight reduction goal. Thus this study showed that self-esteem in massive weight loss patients could be improved by body contouring surgery but did not affect long-term weight maintenance which may require behaviour modification through the team efforts of a weight reduction counselor, therapist, and surgeon.

### 3.7. Menderes et al. [17]

11 patients (7 women and 4 men) who underwent body contouring surgery following vertical banded gastroplasty (VBG) had their self-consciousness assessed by the Derriford Appearance Scale. The questionnaire administered was modified from its original structure, taking into account local norms. The modified questionnaire consisted of 3 subscales:

1. general self-consciousness (GSC) of appearance;
2. social self-consciousness (SSC) of appearance; and
3. sexual and bodily self-consciousness of appearance (SBSC).

The difference between the original questionnaire and the final modified version is given in Table 10.

Each of the patients were asked to answer each of the questions in each subscale with one of five possible statements (from almost never to almost always), and the level of distress was measured with statements (from not at all distressed to extremely distressed) in a Likert type format.

The results are shown in Table 11. As can be seen, there is a significant improvement in GSC and SBSC after bariatric surgery and then after body contouring surgery. SSC also improved to a substantial degree after bariatric surgery and then after body contouring surgery. No statistical analysis was carried out.

### 3.8. Van Der Beek et al. [18]

Assessed quality of life and satisfaction with body contouring surgery in 43 patients who had undergone bariatric surgery. Quality of life was assessed by the Obesity Psychosocial State Questionnaire (OPSQ) (Table 12) which measures seven domains with items in each domain having a five-point rating from 1 (almost never) to 5 (almost always). The questionnaire was administered twice retrospectively following body contouring surgery, the first time to record their prebody contouring quality of life and a second time to record their postbody contouring quality of life. Satisfaction was measured simply by asking the patient about their overall satisfaction with the procedure.

There were improvements in almost all domains with the greatest improvements being in the physical functioning and physical appearance domains (*P* < 0.001). They felt less depressed, more satisfied with their appearance, and felt that they had more control over their eating behaviours following reconstructive surgery (*P* < 0.001). In terms of overall satisfaction with the procedure, sixty-seven percent of patients felt satisfied with the overall result of the operation.

### 3.9. Singh et al. [19]

Applied the SF-36 questionnaire to a total of 104 individuals belonging to four groups of patients including a control group (27 patients), an obese group (31 patients), a postbariatric surgery group (30 patients), and a postbody contouring surgery group (16 patients). The SF-36 questionnaire consists of 8 scales that assess both physical and mental components of health as shown in Table 13.

Comparison of the outcomes for the social functioning and role-emotional components of the SF-36 questionnaire between the postbariatric surgery and postbody contouring groups found a statistically significant reduction in QoL in the postbody contouring group compared to the postbariatric surgery group. However, physical functioning was considerably improved in the postbody contouring group compared to the postbariatric surgery group, although not to a statistically significant degree.

### 3.10. Coriddi et al. [20]

Conducted a prospective telephone survey assessing several functional outcomes and satisfaction before and after body contouring surgery (Table 14). 52 patients who had abdominal contouring procedures (41 had panniculectomies and 11 had abdominoplasties) were recruited with 49 responding to the survey (94% response rate).

Apart from shoulder pain, there were statistically significant improvements in all functional outcomes between the pre- and postbody contouring groups. In terms of satisfaction, 91.8 percent of patients said that they would have their body contouring procedure again or would recommend it to a friend.

### 3.11. Klassen et al. [21]

Used qualitative patient interviews to obtain information regarding quality of life and satisfaction issues in body contouring surgery. Forty-three massive weight loss postbody contouring surgery patients were interviewed. The interviews took the form of patients describing their weight loss journey with probes to explore the impact of obesity, weight loss, and body contouring surgery on their quality of life. Analysis of the interviews revealed a number of important health and aesthetic concerns which were explained in terms of 5 core themes.

1. Appearance-related concerns.
2. Physical health concerns.
3. Sexual health concerns.
4. Psychological health concerns.
5. Social health concerns.

Although no quantitative methods were employed to assess the impact of body contouring surgery on massive weight loss patients, this qualitative study demonstrated that body contouring leads to an overall improvement in the 5 core themes cited above and was an important step in the completion of the entire weight loss process for patients. Furthermore, the issues cited above enable investigators to appreciate QOL issues from the patient's perspective and may provide the conceptual framework necessary to help develop more specific PRO instruments.

## 4. Discussion

Table 15 provides a summary of the instruments used in each study, if the instruments were psychometrically validated, were patient reported, and if they were modified from their original format by the investigators. 

Psychometric validity includes a number of concepts. Some of the essential concepts include:

- content validity—refers to the instrument incorporating relevant questions/sections of interest within it (i.e., how adequately the sampling of items reflects its aims). The questions included within an instrument are usually determined by experts within the field in which the instrument was designed for use;
- construct validity—essentially involves a conceptual definition of the construct to be measured by the instrument and then assessing the internal structure of its components and the theoretical relationship of its item and subscale scores;
- concurrent validity—compares the outcomes of the newly developed instrument with established “gold standard” instruments to determine how well the new instrument purports to measure what it measures in comparison to older, better established instruments;
- predictive validity—this assesses an instrument's ability to predict future outcomes (e.g., resource use or treatment outcomes);
- test-retest reliability—this ascertains the extent of agreement when the same instrument is applied to the same cohort of patients by the same investigator at two different time points;
- interrater reliability—refers to the degree of agreement between outcomes when the same instrument is applied to the same cohort of patients by two or more different investigators;
- sensitivity to change—the extent to which the instrument demonstrates change over time in comparison to “gold standard” measures (e.g., change measured my more established measures);
- acceptability/Feasibility/Utility—this is a measure of how useful and acceptable the instrument is to the investigator and or patient.

In an ideal setting, each PRO instrument would have been validated for every category mentioned above. This would ensure that the instrument measures what it was designed for with a high degree of accuracy. However, this is not always possible or necessary. As long as content validity, construct validity, and predictive validity are satisfactory, the instrument could potentially provide accurate measures of the concept of interest. Table 15 shows that most studies used psychometrically validated instruments while some used measures that were nonpsychometrically validated. A detailed discussion of the psychometric properties of each of the measures employed by the different studies is beyond the scope of this review. However, an important point of note is that all psychometric measures employed by the different studies were not developed to provide outcomes in postbody contouring surgery patients. In other words, none of the psychometrically validated instruments employed within the studies reviewed were developed specifically for the population of interest, that is, postmassive weight loss body contouring surgery patients. Hence, from the outset, content validity of the PRO instruments used is breached. This makes the QoL outcomes obtained from these instruments (in postmassive weight loss body contouring surgery patients) questionable. As a result, this review has identified the need for condition-specific outcome measures. Furthermore, certain studies modified the psychometrically validated measures used. It is hard to predict the impact of modification on each particular instrument, but taking into consideration the type and extent of modification mentioned in each study report, it would be reasonable to assume that the modification may have led to a slight improvement in the validity of the instrument. This is because they were modified to take into account the population of interest, that is, to improve construct validity. For example, the modifications made to the original Derriford appearance scale, a validated outcome instrument, used by Menderes et al. were modified taking into account local norms and patient participation. It is less likely, but not impossible, that the modification might have negatively impacted the validity.

Squires et al. present an advanced protocol for the psychometric assessment of instruments based on the Standards for Educational and Psychological Testing [22]. Validity, reliability, and acceptability are all assessed. A unitary approach to validity consisting of accumulating evidence based on instrument content, response processes, internal structure, and relations to other variables is taken while reliability is assessed with internal consistency coefficients and information functions. Acceptability of the instrument is assessed with missing data frequencies and the time required to complete the survey. Apart from this “traditional” method of assessing psychometric properties, other more modern methods of psychometric assessment have been developed, most useful of which is the Rasch model analysis which was developed by Rasch [23]. Rasch model analysis provides psychometric information that is not provided with the above mentioned more traditional analyses. Rasch model analysis is a probability model that converts the ordinal scores obtained by summing item scores into interval measures [24]. While the ordinal raw scores used in traditional analyses are typically used as if they were interval in nature, the measures produced by Rasch analysis are on an equal-interval scale that is common to both persons and items. *Rasch analysis uses these equal-interval measures to assess multiple psychometric characteristics specific to the population for which the PRO instrument is being developed. This enables the design of PRO instruments which are more accurate and sensitive to clinical changes.*

All measures used were patient reported as indicated in Table 15. The advantage of patient reported measures includes the fact that the individual who was subjected to the intervention who should theoretically have most insight into the impact of the intervention provides the outcome. In addition, in a more general sense, it enables healthcare payers to appreciate the views of patients on particular interventions and may provide a less biased perspective to a particular treatment option. Hence, patient reported outcomes may have more weight when healthcare payers decide to fund a particular procedure.

## 5. Conclusion

As mentioned above, a systematic review of PRO instruments to measure quality of life and patient satisfaction following body contouring surgery identified five PRO measures with varying psychometric validity. However, it must be pointed out that none of these measures have been specifically developed for body contouring surgery patients, and hence the title of the study is a misnomer to its content. Reavey et al. conclude that there is a need to develop specific PRO measures for this group of patients. This review has found a number of studies which have attempted to assess QoL and patient satisfaction in postbody contouring massive weight loss patients with less than ideal PRO measures. Hence, the author agrees that there is an urgent need to develop specific and well constructed PRO instruments in order to obtain reliable information regarding QoL and patient satisfaction following body contouring surgery in MWL patients. Furthermore, future investigators should use newer techniques of assessing psychometric validity of newly developed instruments which are both more convenient and reliable compared to more traditional techniques. 

## Figures and Tables

**Table 1:** Search terms used with the Boolean operation AND to combine terms.

Body contouring		Body image		Massive weight loss/MWL
Abdominoplasty				
Panniculectomy		Psychological function		Postbariatric
Torsoplasty				Weight reduction
Thoracoplasty	AND		AND	
Brachioplasty/arm lift		Quality of life		
Thigh lift				
Reduction mammaplasty/breast reduction		QoL		
Facelift		Psychosocial function		

**Table 2:** Summary of characteristics of studies cited for this review.

Study	Number of patients	Procedure(s)	Control group	Pyschosocial function assessed	Quality of life assessed	Average weight loss following bariatric surgery	Study design: prospective (P)/retrospective (R)	Mean age
Lazar et al. [11]	41 (32 females, 9 males)	Abdominoplasty	No	Yes	Yes	40.2 kg	R	Median age 38 years
Song et al. [12]	18 (16 females, 2 males)	Panniculectomy or abdominoplasty Eleven patients underwent lower body lift or breast reduction or brachioplasty	No	Yes	Yes	138 ± 76 lbs	P	46 ± 10 years
Cintra Jr. et al. [13]	16 (all females)	Abdominoplasty	No	Yes	Yes	23.8 kg/m^2^	R	40.1 ± 8 years
Stuerz et al. [14]	34 (30 females, 4 males)	Abdominoplasty	Yes	Yes	Yes	16 kg/m^2^	P	37.1 ± 9.3 years
Pecori et al. [15]	10 (all females)	Abdominoplasty (7), leg and/or arm lifts (8), torsoplasty (2)	Yes	Yes	No	49 kg	R	Range 28–56 years
Lanier [16]	50 (28 females, 22 males)	Abdominoplasty (24), facelift (18) Reduction mammaplasty (10) Blepharoplasty (8), mastopexy (8), thigh reduction (5), arm reduction (2)	No	Yes	No	126 lbs	R	Not stated
Menderes et al. [17]	11 (7 females, 4 males)	Abdominoplasty (11), reduction mammaplasty (3), lateral thigh lift (2), gynaecomastia (3), medial thigh lift (1), liposuction (3)	No	Yes	No	57.6 kg	R	37.4 years
Van Der Beek et al. [18]	43 (41 females, 2 males)	Primarily abdominoplasty and breast reduction/augmentation	No	No	Yes	51.3 kg	R	41.5 years
Singh et al. [19]	104 (85 females, 19 males)	Unspecified	Yes	Yes	Yes	N/A	R	42
Coriddi et al. [20]	49 (40 females, 9 males)	Panniculectomy and abdominoplasty	No	Yes	Yes	21.5 k/m^2^	P	45.8
Klassen et al. [21]	43 (40 females, 3 males)	Abdominoplasty (31), liposuction (18), Upper arm lift (14), breast lift (10), lift (9), buttock lift (6)	No	Yes	Yes	N/A	R	47

**Table 3:** Psychological evaluation questionnaire and patient answers.

Questions	Yes (%)	Good (%)	No (%)	Bad (%)	Intermediate opinion (%)
How do you feel today?		65.4		15.4	19.2
What is your opinion about abdominoplasty?		50		23.1	26.9
Are the results in accordance with your expectations?	61.5				38.5
Are the scars a problem for you?	61.5		11.6		26.9
Do you like your new body?	53.9		11.5		34.6
Do other people regard you differently now?	61.5		38.5		
Has your daily life improved?	84.6		7.7		7.7
What if abdominoplasty had been contraindicated?		38.5		61.5	
Would you agree to redo abdominoplasty?	96.1		3.9		

**Table 4:** Instruments used by Song et al. [12].

Area of assessment	Outcome measures	General or specifically developed for study
Body perception and ideals	Pictorial body image assessment (PBIA)	Modified version of the Stunkard Silhouette tool developed specifically for this study

Body image satisfaction and areas of distress	(i) Body image and satisfaction assessment (BISA) (ii) Current body image assessment (CBIA)	(i) Specifically developed for this study (ii) Specifically developed for this study

General and condition specific quality of life	(i) Health related quality of life (HR-QoL) (ii) The postbariatric surgery quality of life (PBSQoL) survey	(i) A general instrument to measure QoL (ii) Specifically developed for this study

Mood	Beck's inventory	General instrument to assess mood

**Table 5:** Description of each of the outcome tools employed by Song et al. [12].

Outcome measure	Description
PBIA	A pictorial representation from underweight to severely obese on a 13-point scale on which the patient indicates which one they believe they were before bariatric surgery, their appearance before body contouring surgery, and their personal ideal silhouette

BISA	Divides the body into 10 areas (e.g., thighs, abdomen) with a visual analogue scale from 0, signifying extreme dissatisfaction, to 10, signifying perfect satisfaction. A possible maximum score of 100 and minimum of 0

CBIA	Assesses areas of greatest dissatisfaction. The patient is handed a blank canvass of human outlines representing the front and back views on which they circle up to three areas of distress. These areas are anatomically coded in order to detect changes in areas of distress as patients underwent body contouring

HR-QoL	A modification of the SF-36 questionnaire which is used to assess physical function, self-esteem, sexual function, physical distress, and work function

PBSQoL	A quality of life measure that was specifically designed for the postbariatric weight loss patient population. It assessed areas such as feelings of attractiveness, skin rash and infection, ease of exercise, public embarrassment about loose skin, ease of shopping, and clothing fit

Beck's inventory	A highly sensitive and validated measure of depression symptoms used to assess mood

**Table 6:** Final results of the quality of life questionnaire.

Category	Level	Result (%)
Good adaptation	1	43.8
Mild adaptation	2	37.5
Moderate maladaptation	3	6.3
Severe maladaptation	4	6.3
Very severe maldaptation	5	6.3

**Table 7:** Description of outcome tools used by Stuerz et al. [14].

Outcome measure	Description
Strauss and Appelt's Questionnaire for assessing one's own body	This questionnaire consists of 52 items which are answered with “true” or “not true” and consists of four subscales: (1) Attractiveness/self-confidence (2) Accentuation of external appearance (3) Worry about possible physical defects (4) Problems regarding sexuality

Subscale “Emphasis on attractiveness” of the Body Perception Questionnaire by Paulus	This is a 22-item scale which assesses the extent to which appearance is adjusted to meet social norms

The Life Satisfaction Questionnaire	Covers ten areas of life, is, an index of general life satisfaction, and includes, healthiness, work life, financial status, leisure, partnership, relationship to own children, own person, sexuality, friends/relatives, and residence

The Hospital Anxiety and Depression Scale	A self-rating scale which consists of two separate subscales for anxiety and depression (each with 7 items) and is used to detect states of depression and anxiety

Author's general questionnaire	Developed by the authors of the study and inquires about factors such as financing, expectations, reasons, desire for any other plastic surgery, dealing with the scar, satisfaction, effects on leisure activities, sexuality, inhibitions, and preoperative surgical information

**Table 8:** Description of patient groups in the study carried out by Pecori et al. [15].

Group	Number of patients	Description
OB group	20	Morbidly obese women prior to biliopancreatic diversion (BPD)
POST group	20	>2 years following BPD
POST-A group	10	Postobese women following BPD who required cosmetic procedures
POST-B group	10	Postobese women after BPD and subsequent cosmetic surgery
Control	20	Lean and healthy individuals

**Table 9:** BUT score data in subjects of POST-A and POST-B groups (mean ± SD).

	POST-A	POST-B
Global severity index	1.68 ± 1.13	1.04 ± 0.63
Weight phobia	2.31 ± 1.41	1.80 ± 0.88
Body image concern	2.23 ± 1.54	1.28 ± 0.72
Avoidance	1.07 ± 1.1	0.35 ± 0.55
Compulsive self-monitoring	1.02 ± 0.64	1.09 ± 0.79
Depersonalization	1.09 ± 0.91	0.46 ± 0.56
Positive symptoms total	10.7 ± 7.01	10.1 ± 9.30
Positive index distress	4.10 ± 0.81	4.07 ± 1.37

**Table 10:** Modification of the Derriford Appearance Scale by Menderes et al. [17] leads to fewer questions in each section of the scale.

Derriford appearance scale (DAS-59)	Original no. of questions	No. of questions in modified questionnaire
GSC	17	12
SSC	20	9
SBSC	9	5

**Table 11:** Evaluation of self-consciousness.

	Preoperative perception of SC	Perception of SC between bariatric and plastic surgical interventions	Perception of SC after bariatric and plastic surgery interventions
General self-consciousness	34.3 (3.1)	27.6 (2.5)	21.2 (1.9)
Social self-consciousness	25.1 (2.8)	19.4 (2.1)	16.6 (1.8)
Self-consciousness of appearance	17.7 (3.5)	11.8 (2.3)	8.2 (1.6)

**Table 12:** The Obesity Psychosocial State Questionnaire (OPSQ).

Scales	Example item
Physical function	To kneel or duck easily
Mental well-being	To feel depressed (reversed score)
Physical appearance	To feel fat when someone takes a picture (reverse score)
Social acceptance	To be discriminated because of my weight (reverse score)
Self-efficacy	To feel helpless toward my eating behaviour (reversed score)
Intimacy	To have sexual problems because of my weight (reversed score)
Social network	To visit friends and acquaintances

**Table 13:** The eight scales of the SF-36 which assess physical and mental components of health.

Scale	Measures
Physical component	
Physical functioning	Limitations in physical activities due to health problems
Role-physical	Limitations in usual daily activities due to health problems
Bodily pain	Pain experienced due to health problems
General health	General health perceptions
Vitality	Self-perceived energy and fatigue
Mental component	
Social functioning	Limitations in social activities due to physical or emotional problems
Role-emotional	Limitations in usual daily activities due to emotional problems
Mental health	Perceived well-being and the presence of any psychological distress

**Table 14:** Functional status survey before and after abdominal contouring surgery.

Question*	Before body contouring	After body contouring
Neck pain		
Back pain		
Shoulder pain		
Abdominal pain		
Pain during exercise		
Difficulties with walking		
Difficulties with standing		
Difficulties with posture		
Difficulties with sleeping		
Difficulties with travel		
Difficulties with work tasks		
Difficulties with personal hygiene		
Difficulties with toilet habits		
Difficulties finding clothes		
Lymphedema		
Skin irritation		
Lower extremity paresthesias (numbness/tingling)		
Lower extremity weakness		

Question^†^	Before body contouring	After body contouring

Ability to climb stairs		
Ability to descend stairs		
Ability to jog/run		
Ability to rise from a squatting position		
Ability to play with kids		
Ability to do household tasks		

Satisfaction^‡^		

Would you have this procedure again? yes/no		
Would you recommend this procedure to a friend? yes/no		

*Respondents reported on a 10-point Likert scale ranging from 1 (infrequent) to 10 (often).

^†^Respondents reported on a 10-point Likert scale ranging from 1 (completely able) to 10 (unable).

^‡^Respondents reported on a 10-point Likert.

**Table 15:** Summary of instruments employed in each study, psychometric validity, modification by investigators, and if they were patient reported outcome measures.

Study	Questionnaire/s employed	Psychometrically validated?	Modified for study?	Patient reported?
Lazar et al. [11]	(1) Three-item subjective questionnaire	(1) No	(1) N/A	(1) Yes
	(2) Nine-item psychological status questionnaire designed by trained psychologist	(2) No	(2) N/A	(2) Yes

Song et al. [12]	(1) Pictorial body image assessment (2) Body image and satisfaction assessment (3) Current body image assessment (4) Health related quality of life (5) The postbariatric surgery quality of life survey (6) Becks inventory	(1) No (2) No (3) No (4) Yes (5) No (6) Yes	(1) N/A (2) N/A (3) N/A (4) Yes (5) N/A (6) No	(1) Yes (2) Yes (3) Yes (4) Yes (5) Yes (6) Yes

	(1) Strauss and Appelt's questionnaire (for assessing body image)	(1) Yes	(1) No	(1) Yes
Stuerz et al. [14]	(2) “Emphasis on attractiveness” subscale of the Body Perception Questionnaire by Paulus	(2) Yes	(2) Yes	(2) Yes
	(3) The life satisfaction questionnaire	(3) Yes	(3) No	(3) Yes
	(4) The hospital anxiety and depression scale	(4) Yes	(4) No	(4) Yes
	(5) Author's general questionnaire	(5) No	(5) N/A	(5) Yes

Cintra et al. [13]	Adaptive Operationalized Diagnostic Scale	Yes	No	Yes

Pecori et al. [15]	Body uneasiness test	Yes	No	Yes

Lanier [16]	2 questions devised by investigators	No	N/A	Yes

Menderes et al. [17]	Derriford appearance scale	Yes	Yes	Yes

Van Der Beek et al. [18]	The obesity psychosocial state questionnaire	Yes	No	Yes

Singh et al. [19]	SF-36	Yes	No	Yes

Coriddi et al. [20]	(1) Functional status survey (2) Two Question Satisfaction survey	No	N/A	Yes

Klassen et al. [21]	Qualitative interview	N/A	N/A	Yes
